# The Effect of Docetaxel (Taxotere^®^) on Human Gastric Cancer Cells Exhibiting Low-Dose Radiation Hypersensitivity

**DOI:** 10.4137/cmo.s463

**Published:** 2008-03-28

**Authors:** Elizabeth K. Balcer-Kubiczek, Mona Attarpour, Jian Z. Wang, William F. Regine

**Affiliations:** 1 University of Maryland School of Medicine, Department of Radiation Oncology, Baltimore, MD 21201, U.S.A; 2 University of Maryland Marlene and Stewart Greenebaum Cancer Center, Baltimore, MD 21201, U.S.A; 3 The Ohio State University College of Medicine, Department of Radiation Medicine, Columbus, OH 43210, U.S.A

**Keywords:** docetaxel, low-dose radiation hypersensitivity, G_2_ checkpoint, ATM, Chk1, Cdc25A, Cdk1, H3, cyclin B1

## Abstract

Low-dose radiation hypersensitivity (HRS) describes a phenomenon of excessive sensitivity to X ray doses <0.5 Gy. Docetaxel is a taxane shown to arrest cells in the G_2_/M phase of the cell cycle. Some previous studies suggested that HRS might result from the abrogation of the early G_2_ checkpoint arrest. First we tested whether HRS occurs in gastric cancer—derived cells, and whether pre-treatment of cells with low docetaxel concentrations can enhance the magnitude of HRS in gastric cancer cells. The results demonstrated HRS at ~0.3 Gy and the synergy between 0.3 Gy and docetaxel (3 nM for 24 h), and the additivity of other drug/dose combinations. The synergistic effect was associated with a significant docetaxel-induced G_2_ accumulation. Next, we evaluated in time-course experiments ATM kinase activity and proteins associated with the induction and maintenance of the early G_2_ checkpoint. The results of multi-immunoblot analysis demonstrate that HRS does not correlate with the ATM-dependent early G_2_ checkpoint arrest. We speculate that G_2_ checkpoint adaptation, a phenomenon associated with a prolonged cell cycle arrest, might be involved in HRS. Our results also suggest a new approach for the improvement the effectiveness of docetaxel-based radiotherapy using low doses per fraction.

## Introduction

Worldwide, gastric cancer is the fourth most common malignancy and the second most fatal (Alberts et al. 2003; Edwards et al. 2006). Significant geographic variation exists with high-risk areas including Japan and Europe, and low risk areas including the United States (Alberts et al. 2003). Despite its relatively low incidence in the United States, gastric cancer is a significant cause of morbidity and mortality, with 23,000 cases per year, resulting in 13,000 deaths. At time of diagnosis, approximately 60% to 75% patients with gastric cancer have advanced disease with a five-year survival rate ranging from 3% to 22%, depending on the extent of the disease (Edwards et al. 2006). The available clinical data in the treatment of gastric carcinoma have demonstrated that radiation therapy has a role for improving local control and, in combination chemotherapy, survival (Alberts et al. 2003; Edwards et al. 2006; Das and Ajani, 2005).

Several drugs belonging to different classes have been proven active in patients with gastric cancer (Alberts et al. 2003; Das and Ajani, 2005). Clinical results have been reported for antimetabolites, platinum-based agents, DNA topoisomerase inhibitors and more recently taxanes. However, about half of patients are resistant to chemotherapy. Another limitation is cumulative, systemic toxicity after typical prolonged and high-dose drug therapy that often compromises “full-dose” therapy (Alberts et al. 2003; Das and Ajani, 2005; Oehler and Ciernik, 2006). The role of standard radiotherapy in the treatment in gastric cancers remains controversial because of the marked radiation sensitivity of neighboring organs (Oehler and Ciernik, 2006). A great deal of interest has focused on the search of new strategies to enhance the anticancer effects of lower drug and/or radiation doses.

A recent multiinstitutional Phase I study evaluated the efficacy of low dose fractionated radiation therapy (0.6 Gy *per* fraction, two fractions *per* day) in combination with gemcitabine in patients with gastrointestinal cancers (Regine et al. 2007). This successful strategy for the delivery of upper abdominal radiation has been based on experimental data demonstrating the low dose radiation hypersensitivity (HRS) phenomenon i.e. a statistically significant disagreement between predictions of the linear-quadratic model and measurements of cell survival after radiation doses of less 1 Gy (Marples and Joiner, 1993; Short and Joiner, 1998; Short et al. 1999; Joiner et al. 2001). According to these reports, the linear-quadratic model (Kellerer and Rossi, 1972) overestimates survival in the low dose range (Joiner et al. 1993). Studies with cells in specific cell cycle phases demonstrated that HRS response is more prominent in G_2_/M phase cells, compared to that in the asynchronous population (Marples et al. 2003; Short et al. 2003). It has been proposed that enhanced sensitivity of G_2_/M phase cells to low radiation doses is associated with the abrogation of the *early* G_2_ checkpoint responses including a failure to delay entry into mitosis and to initiate DNA repair (Marples et al. 2003; Short et al. 2003). The early G_2_ checkpoint is ATM dependent, specific to cells irradiated at G_2_ and transient, resolving within 1 h after irradiation. A hallmark of the early G_2_ checkpoint is a rapid reduction in mitotic index (Xu et al. 2001, 2002). The *late* G_2_ checkpoint is activated in cells irradiated in G1 and S, is ATM independent and sustained, and begins to manifest only several hours after irradiation. A hallmark of the late G_2_ checkpoint is an accumulation of cells in G_2_. The late G_2_ checkpoint has not been directly implicated in HRS responses.

4-acetoxy-2α-benzoyloxy-5β, 20-epoxy-1, 7β, 10β-trihydroxy-9-oxotax-11-ene-11α-(2R, 3S)-3-*tert*-butoxycarbonylamino-2-hydroxy-3-phenyl-propionate (RP 56976A; docetaxel) is a microtubule-stabilizing taxane, which has recently been approved for use in the clinic for the treatment of advanced gastric malignancies (Das and Ajani, 2005). Preclinical studies demonstrated that docetaxel is active against gastric cancer cells as a single agent or in combination with antimetabolites or radiation (Ricotti et al. 2003; Balcer-Kubiczek et al. 2006). The biological rationale for combination therapy with docetaxel has been based on the cell cycle effects of the drug, specifically its well-established ability to accumulate cells at the G_2_/M phase (Ricotti et al. 2003; Abal et al. 2003; Morse et al. 2005; Hernández-Vargas et al. 2007). The primary mechanism of docetaxel action is mitotic spindle damage (reviewed by Abel et al. 2003). Recent mechanism-of-action studies demonstrated different mitotic responses according to drug concentration (Ricotti et al. 2003; Abal et al. 2003; Morse et al. 2005; Hernández-Vargas et al. 2007). High clinically irrelevant concentrations induce a permanent mitotic arrest (Ricotti et al. 2003; Abal et al. 2003; Hernández-Vargas et al. 2007). By contrast, clinically relevant concentrations interfere with mitotic progression by transiently activating the spindle checkpoint, without significantly arresting cells at M-phase (Abal et al. 2003; Morse et al. 2005; Balcer-Kubiczek et al. 2006; Hernández-Vargas et al. 2007).

Against this background, we initiated a pre-clinical study to determine whether HRS occurs in gastric cancer—derived cells, and whether pre-treatment of cells with low docetaxel concentrations can enhance the magnitude of HRS response. In addition, we evaluated six proteins associated with the induction and maintenance of the early G_2_ checkpoint. The radiation-induced G_2_ DNA damage checkpoint operates at least in part by maintaining phosphorylation of the checkpoint protein kinase 1 (Chk1), a modification that prevents the cycle division cycle 25C (Cdc25C) phosphatase from activating the cyclin-dependent protein kinase 1 (Cdk1; also called Cdc2) (Furnari et al. 1997; Sanchez et al. 1997; Peng et al. 1997). An initiating event for this pathway is transient phosphorylation of ataxia telangiectasia mutated (ATM) protein on serine (S) 1981 (Bakkenist and Kastan, 2003). The inability of cells to maintain G_2_ checkpoints correlates with the inability to maintain phosphorylations of S317 on Chk1, S216 on Cdk25C, tyrosine (Y) 15 on Cdk1 (Sanchez et al. 1997; Peng et al. 1997; Gatei et al. 2003; Syljuåsen et al. 2003). We completed our evaluation of the G_2_ checkpoint by assessing the expression of cyclin B1 and phosphorylation of S10 in histone H3. S10 phosphorylation begins late in G_2_, is complete in prophase and absent during the anaphase/telophase transition (Juan et al. 1998; Prigent and Dimitrov, 2003). Cyclin B1 is expressed throughout mitosis (Juan et al. 1998). Cyclin B1 and/or H3 S10 were previously used to identify mitotic cells after various treatments (Juan et al. 1998; Xu et al. 2002; Marples et al. 2003; Deckbar et al. 2007). Phosphorylation of ATM S1981, Chk1 S317 or Cdk1 expression were studied previously in the context of molecular effects of low radiation doses including HRS (Marples et al. 2003; Bakkenist and Kastan, 2003; Enns et al. 2004; Buscemi et al. 2004; Short et al. 2005; Deckbar et al. 2007).

## Materials and Methods

### Cell lines and cell culture

Three cell lines tested for the presence of HRS were established from the following tumors: untreated gastric adenocarcinoma (AGS), non-small cell lung adenocarcinoma (A549) and androgen-independent prostate carcinoma (PC3) (obtained from the American Type Culture Collection, Manassas, VA, U.S.A.). HRS was demonstrated previously in the A549 and PC3 cell lines (Joiner et al. 2001; Enns et al. 2004); in the present experiments these cell lines served as positive controls for our clonogenic assay methodology. Cells were grown as attached monolayers in the F12 Kaighn’s medium supplemented with 10% fetal bovine serum and without antibiotics.

### Docecetaxel treatment

Docetaxel (Taxotere^®^; lot 0002820; MW = 807.9 g/mol) obtained from Aventis Pharmaceuticals, a member of sanofiaventis Group (Bridgewater, NJ, U.S.A.) in a pure crystalline powder form was stored in sterile dimethylsulfoxide (DMSO); 99.9% pure; Sigma; St. Loius, MO, U.S.A.) as 100 μg/ml solution at −20 °C. Stock solution was diluted to the required concentrations in the nM-range by successive dilutions in DMSO and in growth medium; similarly diluted DMSO was used to carry out mock drug exposure (Balcer-Kubiczek et al. 2006). Drug treatments with or without subsequent X-irradiation were performed as in previous studies (Balcer-Kubiczek et al. 2006).

### X-irradiation

Cells were irradiated with single doses between 0.05 and 6 Gy using a Pantak machine (250 kV, 13 mA with a 0.5-mm Cu + 1-mm Al filter) producing X-rays at a dose rate of 0.3 Gy min^−1^ (SSD = 82 cm) or 2.4 Gy min^−1^ (SSD = 32 cm). The lower dose rate was used for doses < 0.5 Gy.

### Survival experiments with graded doses of X-rays

To test for the presence of HRS, stock cultures of AGS, A549 and PC3 cells were established two days before X-irradiation, then dissociated using 0.25% trypsin/0.05% EDTA solution, counted electronically and diluted to the required concentrations depending on the expected surviving fraction after irradiation. Our previously published AGS, A549 and PC3 cell survival curves were used to plan dilutions for groups to be irradiated with doses >1 Gy (Balcer-Kubiczek et al. 1999, 2006, 2007). A surviving fraction of 1 was assumed for groups to be irradiated with doses ≤1 Gy (i.e. equal cell numbers were plated in control and dose groups). Three to five 100-mm culture dishes were plated from subculture for each dose. Dishes were incubated overnight at 37 °C prior to X-irradiation for exponential growth recovery. Following irradiation, cells were returned to incubators for 14–21 days to allow full development of surviving colonies in all dose groups. The growth medium was renewed weekly. At the end of incubation period colonies were stained with 1% crystal violet in ethanol, and manually counted for surviving fraction determinations by a standard colony formation assay (Puck and Marcus, 1956). Up to 15 replicate experiments were performed at doses ranging from 0.05 to 10 Gy

### Survival experiments with single X-ray doses alone or in combination with docetaxel

To test for interaction between docetaxel and X-irradiation, AGS cells were plated as above. After overnight incubation the medium was removed and replaced with the medium containing 0 (<0.05% DMSO) or 3 nM docetaxel. Cells were irradiated with 0, 0.3 or 2 Gy of X-rays at 0, 4 or 24 h after the initiation of drug treatment. Immediately (<10 min) following X-irradiation cells were returned to normal drug-free growth medium, as described before (Balcer-Kubiczek et al. 2006). Clonogenic survival was determined as described above in the previous section. Replicate experiments, each comprising of 18 experimental groups (i.e. three time points *times* three X-ray doses with 0 or 3 nM docetaxel), were performed 15 times.

### Statistical analysis of survival data

Survival data sets for each of the three cell lines tested for the presence of low-dose HRS were fitted to the basic two-parameter linear-quadratic (LQ) model (Kellerer and Rossi, 1972) as well as to the four-parameter induced-repair (IR) model (Joiner et al. 1993); for the explicit equation and interpretation of IR model parameters see Short et al. (1999). The LQ or IR model best-fit parameters in Table 1 were obtained using Sigma-Plot software, version 6.10 (SystatSoftware, Inc. San Jose, CA, U.S.A.) and, independently, using JPM^®^ SAS software (Cary, NC, U.S.A.) (data not shown). The two non-linear least-squares regression routines utilize different iterative methods (the Marquardt-Levenberg algorithm in SigmaPlot or the Gauss-Newton algorithm in JPM^®^ SAS software). HRS was judged to be present if the ratio of the survival curve slope measured at low doses (α_s_) to the slope extrapolated from the high-dose response (α_r_) was statistically different from one and the dose (d_c_) at which a local survival minimum occurs was statistically different from zero (9). Graphical presentation of IR or LQ equations with SigmaPlot best-fit parameters for each of the three cell lines was obtained using Physics Analysis Workstation software (CERN Program Library; CERN, Geneva, Switzerland; http://paw.web.cern.ch/paw/).

In combined experiments, the surviving fractions after different radiation doses were normalized to the toxicity of docetaxel when given alone, as described previously (Balcer-Kubiczek et al. 2006). At each time point the survival fraction after radiation dose without docetaxel was compared to the normalized surviving fraction after combined treatment with docetaxel. The two survival values were statistically compared using analysis of variance with subsequent application of Student’s t-test. A more-than-additive effect was judged to be present if the surviving fraction measured after X-ray alone was greater then that measured after combined treatments. The required calculations were performed using PSI-Plot software (Polysoftware International, Salt Lake City, UT, U.S.A.).

### Cell cycle analysis

AGS cells were exposed to docetaxel in 25-cm^2^ flasks at a density of 5 × 10^3^ cells cm^−2^ after overnight incubation. To establish the effect of drug concentration on cell cycle distribution, cells were exposed to various docetaxel concentrations ranging from 0 to 10 nM for 24 h (data not shown). Data from drug dose-response experiments together with the previously established docetaxel cytotoxicity data (Balcer-Kubiczek et al. 2006) were used to design experiments on the dependence of cell cycle parameters on drug exposure durations (described in the figure legend) at 3 nM. Treated and control cells were prepared for flow cytometry analysis by staining with propidium iodine (PI) using reagents and procedures in the Vermont Cancer Center protocol (University of Vermont, Burlington, VT, U.S.A.; http://www.vermontcancer.org/research/cores/flow). Cells were then analyzed for red fluorescence (PI) using Becton Dickenson FACScan machine, as described previously (Balcer-Kubiczek et al. 2007). For each sample, 10,000 events were collected and analyzed. Samples were run in triplicate and experiments repeated three times. Relative G_1_, S, and G_2_/M populations expressed as percentages of the total using the MODFIT computer program version LT3-1 (Verify Software House, Topsham, ME, U.S.A.), as previously (Balcer-Kubiczek et al. 2007).

### Mitotic index assessment

In the process of harvesting AGS cells for flow cytrometry analysis at each time point, aliquots of the cell suspensions were taken and processed for assessment of mitotic activity by fluorescence microscopy. The mitotic index was determined by staining with 4′, 6-diamidino-2-phenylindole (DAPI; Sigma Immunochemicals, St. Louis, MO, U.S.A.), as previously described (Balcer-Kubiczek et al. 2006). In this approach, only chromatin stains with DAPI (at 1.5 μg/ml) fluorescing blue. DAPI-stained cells were scored for morphological evidence of mitosis (300–500 random cells *per* slide, 1500–2000 cells total), as described previously (Balcer-Kubiczek et al. 2006). In each of three independent experiments, two different observers evaluated the same slide and the results were averaged.

### Multi-immunoblot analysis of phosphoproteins

Whole lysate protein samples were prepared from AGS cells after treatment with docetaxel (3 nM for 24 h) or at various time intervals (described in the figure legend) following 0.3 or 2 Gy using reagents and protocols provided by Kinexus Bio-informatics Corporation (Vancouver, BC, CA; http://www.kinexus.ca/kinetworks.htm). Total protein was prepared as described previously (Balcer-Kubiczek et al. 1999). Briefly, cells were washed with ice-cold PBS, scrapped in lysis buffer supplemented with protease and phosphatase inhibitors and sonicated for 15 s. Cell debris was removed by centrifugation at 100,000 rpm for 30 min at 4 ^o^C. Total protein concentrations in each sample were determined by the Bio-Rad protein assay according to the manufacturer’s instructions (Bio-Rad Laboratories, Richmond, CA, U.S.A.). For the Kinetworks™ protein phosphorylation analysis, 25 μg of total protein *per* lane was resolved on a 13% sodium dodecyl sulfate poly-acrylamide gel, transferred to nitrocellulose membrane and probed with a custom mixture of ^32^P–labeled antibodies against cyclin B1, the target phosphoserines on ATM, Chk1, Cdc25C or H3 and phosphotyrosine on Cdk1 (described in the introduction). The Kinetworks™ protein phosphorylation screens and the data analysis were performed by Kinexus Bioinformatics Corporation (Vancouver, BC, CA). The reproducibility of these screens was within 15%. Detailed information on the Kinetworks™ multi-immunoblot analysis has been published (Pelech, 2004; Oh et al. 2007).

## Results

### Single dose-response curves

**Figure 1:**
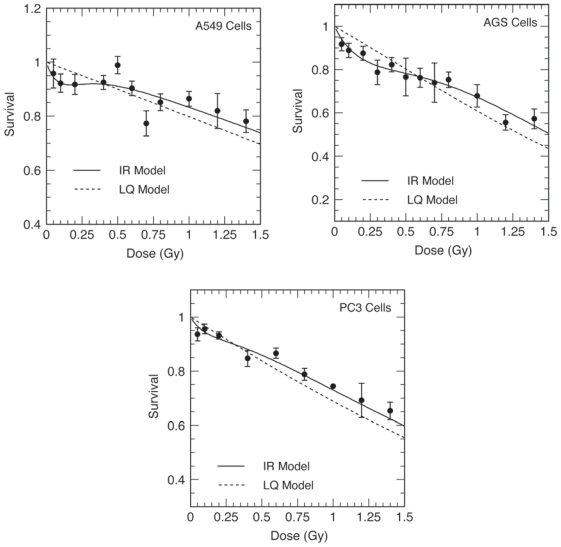
The low-dose portions of the clonogenic survival curves (0.05–1.5 Gy) for human non-small cell lung (A549), gastric (AGS) or prostate (PC3) cancer-derived cells X-irradiated with single doses between 0.05–10 Gy. *Solid line*: the least-square fit to the induced repair (IR) model; *Broken line*: the least-square fit to the linear-quadratic model. IR or LQ model parameters based on the full-dose range (0.05–10 Gy) data are listed in Table 1 *Points*: the mean surviving fractions; *Bars*: standard errors of the mean from 12–15 replicate experiments.

Cell survival was measured for X-ray doses up to 10 Gy using the colony-formation end point. The IR and LQ fits to low dose data are depicted in Figure 1. Fitting the full dose-range survival data using the IR model resulted in best-fit model parameters shown in Table 1. By the criteria described in Materials and Methods (i.e. α_s_/α_r_ ≠ 1 and d_c_ ≠ 0) the AGS gastric and A549 lung adenocarcinoma cell lines exhibited robust HRS, whereas the PC3 prostate cancer cell line did not. Our estimate of the d_c_ dose of 0.11 ± 0.03 Gy for A549 cells agrees very well with the recent estimate of d_c_ of 0.10 to 0.18 cGy for this cell line (Enns et al. 2004). The HRS magnitude as judged by the slope ratio α_s_/α_r_ for A549 cells (Table 1) was 2–3 times greater than the previously reported value of 4 to 5 (Joiner et al. 2001). The d_c_ dose of 0.30 ± 0.05 Gy was calculated for the AGS cell line. The HRS status of the PC3 cell line remains uncertain since the contradictory results were obtained in the present *vs* Gray Laboratory studies (Joiner et al. 2001). Table 1 also shows the values of surviving fractions at 2 Gy (SF_2_) as well as best-fit parameters of the LQ model. SF_2_ is a measure of radiation sensitivity. By this criterion, HRS positive cell lines significantly differed in the intrinsic sensitivity, with A549 cells being two-fold more radioresistent than AGS cells. Thus, our results with HRS positive cell lines are consistent with the previously suggested correlation between SF_2_ and α_s_/α_r_ (Joiner et al. 2001; Mothersill et al. 2002).

### Time course of docetaxel-induced effect on AGS cell cycle distribution

**Figure 2:**
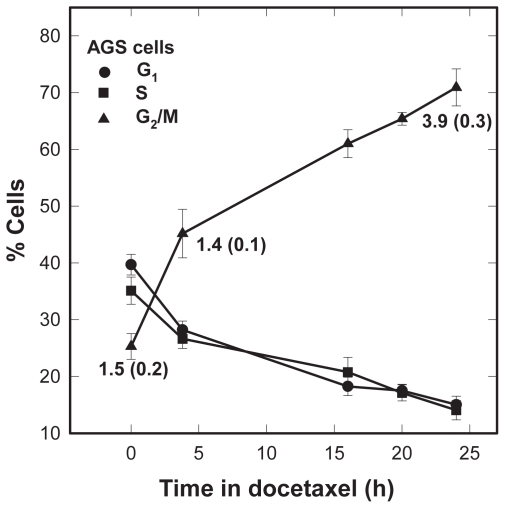
Changes in cell cycle distribution of the AGS gastric cancer cells in response to 3 nM docetaxel treatments over times between 4–24 h. *Numbers within the figure*: the mean mitotic index (standard error) by scoring of mitotic figures. Data points and bars represent, respectively, means and standard errors of triplicate samples analyzed by flow cytometry.

Our preceding experiments showed that following a 24-h treatment, the percentage of AGS cells in the G_2_/M phase increased from ~20% to 75% with docetaxel concentrations up to ~3 nM and reached a near-plateau at docetaxel concentrations >3 nM. Based on this observation and our previous docetaxel cytotocity data for AGS cells (Balcer-Kubiczek et al. 2006) we selected 3 nM for time course studies of cell kinetics. Four hours after the beginning of treatment at 3 nM the proportion of AGS cells in G_2_/M increased approximately twofold and by 24 h ~71% of the cells were in G_2_/M, compared to un-treated control cells (Fig. 2). The G_2_/M arrest was not complete, however, because the remaining ~30% of the cells were approximately equally distributed between the S- and G_1_-phases (~14% and ~15%, respectively). The direct counts of mitotic figures in the samples concurrently analyzed by flow cytometry (numbers in Fig. 2) demonstrated a modest increase of mitotic cells among cells with a G_2_/M DNA content (mitotic index of ~3% following 3 nM docetaxel for 24 h, compared to ~1.5% in non-treated control cells. This result indicates substantial enrichment in G_2_ at various time points after 3 nM of docetaxel.

### Effect on AGS cells of X-irradiation (0.3 or 2 Gy) without or with docetaxel pre-treatment

**Figure 3:**
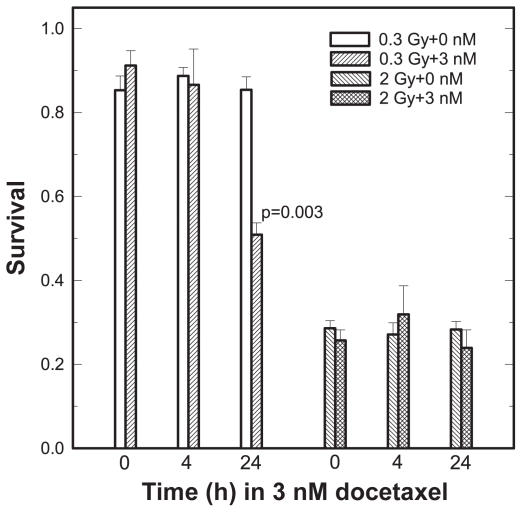
Surviving fractions following X-irradiation plus solvent (0 nM) and X-irradiation after 4-h or 24-h treatment with docetaxel (3 nM). Dividing the surviving fraction for docetaxel at zero X-ray doses normalized the docetaxel plus X-ray surviving fractions. Replicate experiments, each comprising of 18 experimental groups (i.e. three time points *times* two X-ray doses ± 3 nM docetaxel), were performed 15 times.

To test whether the HRS magnitude is influenced by the proportion of AGS cells in G_2_, we pre-treated cultures with 3 nM docetaxel for 0, 4 or 24 h and measured clonogenic survival for docetaxel alone or in combination with 0, 0.3 or 2 Gy X-rays given immediately at the end of a docetaxel time course. Docetaxel toxicity increased with time in the drug at 3 nM and resulted in surviving fractions of 0.98 ± 0.02 at 0-h, 0.36 ± 0.04 at 4-h and 0.014 ± 0.005 at 24-h time points. Within the same experiment, two additional drug-exposed groups were irradiated with 0, 0.3 or 2 Gy over a drug-time course and assayed for survival. The combined –treatment normalized data (see Materials and Methods) and X-ray only data are shown in Figure 3. There are two points to note. First, the X-ray-only data in Figure 3 independently confirmed and extended the initial AGS data in Figure 1. Based on the IR model parameters for the AGS cell line in Table 1, the predicted survival fraction at 0.3 Gy was 0.82 *vs* 0.86 ± 0.08, a weighted mean of surviving fractions at 0.3 Gy measured at 0, 4, 24 h time points. Second, referring next to the X-ray *plus* docetaxel survival data, 0.3 Gy X rays preceded by docetaxel pre-treatment for 24 h produced a robust synergistic effect (p = 0.003, one tail t-test) whereas all the other conditions produced an additive effect between docetaxel and radiation (p > 0.3, one-tail t-test).

### Activation of the ATM kinase and G_2_ checkpoint proteins by radiation and docetaxel in AGS cells

**Figure 4:**
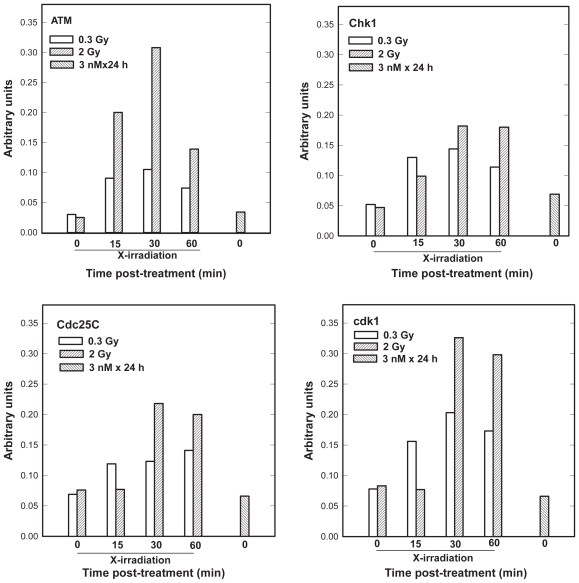
Time-course for the activity of the human ATM, Chk1 or Cdk1 protein kinases and the activity of Cdc25C protein in AGS cells following 0.3 or 2 Gy X-ray dose or immediately after 24-h exposure to 3 nM docetaxel. Shown are the data from one multi-immunoblot experiment. The reproducibility of these protein screens is typically to within 15%.

To test whether ATM and an ATM signaling pathway play a role in HRS we compared early responses of ATM and G_2_ checkpoint proteins to the HRS dose of 0.3 Gy *vs* 2 Gy as a function of time. In view of the observed synergy between docetaxel and radiation we also investigated ATM and G_2_ checkpoint proteins after a prolonged 24-h docetaxel exposure at 3 nM. We first describe the ATM/Chk1/Cdc25C/Cdk1 pathway (Fig. 4). The data in Figure 4 were normalized to the total activity in each lane to emphasize temporal effects and to facilitate comparisons across the protein data sets. Both 0.3 Gy and 2 Gy induced ATM phosphorylation on S1981 that peaked at 30-min and remained elevated 1-h after irradiation. Compared to control values, peak induction levels were a 3- or 10-fold at 0.3 or 2 Gy, respectively. We found that phosphorylation of Chk1 was 2- to 3-fold higher in the two dose groups, compared to controls, and maintained for up 1 h. Similar phosphorylation patterns were observed in responses of Cdc25C and Cdk1 (Fig. 4). Exposure to docetaxel had no effect on the kinase activity of the ATM, Chk1, Cdc25C or the expression of Cdk1.

**Figure 5:**
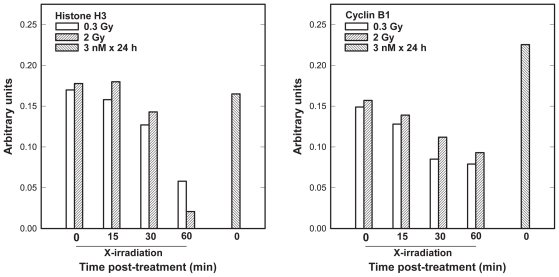
Time-course for the expression of the human histone H3 serine 10 or cyclin B1 protein in AGS cells following 0.3 or 2 Gy X-ray dose or immediately after 24-h exposure to 3 nM docetaxel. Shown are the data from one multi-immunoblot experiment. The reproducibility of these protein screens is typically to within 15%.

To further evaluate G_2_ checkpoint, we assessed markers of mitotic cells, i.e. the degree of H3 his-tone phosphorylation and expression of cyclin B1 (Fig. 5). While phosphorylation of Chk1, Cdc25C and Cdk1 continued to rise during the 1 h post-irradiation period, a decrease in mitotic activity in the two dose groups was evident only at a 1-h time points. Collectively, the data in Figure 4 and 5 are consistent with transient early G_2_ checkpoint responses to 0.3 or 2 Gy of X rays. The duration of this G_2_ checkpoint was weakly dose-dependent. In the case of docetaxel, no correlation was observed between the expression of cyclin B1 and the degree of H3 phosphorylation (Fig. 5).

## Discussion

We have identified the gastric cancer-derived AGS cell line as HRS positive with the d_c_ dose of ~0.3 Gy and demonstrated the combination of docetaxel and 0.3 Gy X-rays is synergistic. This finding distinguishes low radiation dose chemoradiation with docetaxel from conventional chemoradiation therapy since docetaxel produces an additive effect with higher (>1 Gy) radiation doses, as exemplified by the 2 Gy data (Fig. 3). The docetaxel concentration of 3 nM corresponds to plasma levels of docetaxel out to 24–72 h after the end of the dose and administration schedule used in routine clinical practice i.e. 60–100 mg/m^2^ given over 1 h by infusion (Gustafson et al. 2003; Andersen et al. 2006).

Although HRS varied among the three lines tested, the maximum low-dose hypersensitivity was observed in the dose range of ~0.1 –0.3 Gy (Table 1, Fig. 1), in agreement with previous reports (Joiner et al. 2001; Chandna et al. 2002; Short et al. 2003; Enns et al. 2004; Short et al. 2005); however, our estimates of the IR model parameters for A549 or PC3 cells (Table 1) differ from those in previous reports (Joiner et al. 2001). This may be due in part to different techniques used to determine cell survival. An important methodological point about our data is that we used the standard colony-forming assay; by this approach survival curves can be determined down to several logs of cell killing. In contrast, the majority of previous HRS studies used their own laboratory-specific methods including the cell sorter-based plating technique combined with micro-image analysis to quantify colony formation (Short et al. 1998) or the single-cell gel micro-encapsulation technique combined with flow cytometry to quantify proliferation of irradiated cells (Bogen et al. 2001; Enns et al. 2004). One disadvantage of these approaches is that cells are subjected to chemical and/or physical stress that may influence survival results in the low-dose range. In addition, the use of these assays concentrates on the first log of cell killing and high-dose portions of survival curves are obtained by different methods (Short et al. 1998) or not established (Enns et al. 2004). In the latter case the four IR model parameters cannot be obtained with any statistical certainty, as only a few low-dose survival data are available.

We provided circumstantial evidence (Fig. 3) that G_2_/M cells are more sensitive to low radiation doses than exponentially growing cells, because we observed an enhancement of HRS in the AGS cell populations partially synchronized at G_2_ phase (Fig. 2). To within statistical uncertainties enrichment for G_2_ cells by 3 nM docetaxel for 24 h (71% ± 4%) was similar to that by flow cytometry-based phase sorting in the Short study (confidence limits 74%–85%) (Short et al. 2003). Referring again to Figure 3, a 4-h drug exposure resulted in ~40% G_2_ enrichment but had no effect on the magnitude of HRS. We speculate that the duration of cell holding at G_2_ is a more critical variable than the actual proportion of cells at G_2_ at time of irradiation. Our interpretation is based on checkpoint adaptation, a phenomenon in which cells arrested in a checkpoint eventually override this arrest and re-enter the cell cycle despite the fact they have not repaired the damage that elicited the arrest. Adaptation to the G_2_ checkpoint has been documented to occur spontaneously as well as in a response to several forms of DNA damaging agents, including ionizing radiation (Syljuåsen et al. 2003; Deckbar et al. 2007), and prolonged administration of docetaxel at low concentrations (Abal et al. 2003; Morse et al. 2005; Balcer-Kubic-zek et al. 2006; Hernández-Vargas et al. 2007). A 2-fold increase in mitotic index after 24 h in docetaxel (Fig. 2) is consistent with G_2_ checkpoint release despite the presence of damage to the mitotic apparatus; no effect of adaptation is seen in our 4-h data (mitotic indices 1.5% ± 0.2% vs 1.4% ± 0.1%, at 0 or 4-h in docetaxel, respectively. The three studies (Chandra et al. 2002; Short et al. 2003; Enns et al. 2004) reported conflicting effects of cell cycle on HRS, but no experimental details were provided to permit the analysis of these findings in terms of checkpoint adaptation.

The data in Figures 4 and 5 are inconsistent with the hypothesis that enhanced sensitivity of G_2_/M phase cells to low radiation doses is associated with the abrogation of the early G_2_ checkpoint (Marples et al. 2003). Rather our data suggest a dose-dependent transient checkpoint response in G_2_ followed by mitotic delay shortly (<1h) after irradiation of HRS-positive AGS cells. Similar conclusions were published recently (Enns et al. 2004; Short et al. 2005). X-irradiation of AGS cells led to concurrent activation of ATM and three additional proteins (Chk1, Cdc25C and Cdk1) thought to play critical roles in the prevention of premature mitosis. Our data are in agreement with those that have correlated phosphorylation of these proteins with DNA damage-induced G_2_ checkpoint (Fournari et al. 1997; Sanchez et al. 1997; Peng et al. 1997; Short et al. 2005). In addition, our data are consistent with the hypothesis that DNA double-strand breaks (DSBs) are lesions activating the ATM kinase activity followed by ATM-dependent modifications of G_2_ checkpoint proteins (Bakkenist and Kastan, 2003; Buscemi et al. 2004; Deckbar et al. 2007). X-ray doses of 0.3 or 2 Gy induce DSBs *per* cell respectively, whereas docetaxel at low concentrations does not induce DSBs (Rothkamm and Löbrich, 2003, our data not shown). The mitotic cell markers commonly used to monitor entry into mitosis, the expression of H3 S10 and cyclin B1, declined as a function of time after X-irradiation, but the effect was less pronounced at 0.3 Gy than at 2 Gy (Fig. 5). This is the opposite of the Marples findings (Marples et al. 2003) that show no dose-dependent decrease in mitotic indices after radiation exposure <0.6 Gy, i.e. a dose threshold for G_2_ checkpoint activation in HRS positive cell lines. The data supporting this conclusion were obtained 2-h post-irradiation, which is past the duration of the early G_2_ arrest in the majority of ATM-positive cell lines (Xu et al. 2001, 2002). With regard to the effect of docetaxel on the cell cycle distribution (Fig. 5), a 2-fold increase in the expression of cyclin B1 following 24-h docetaxel treatment vs control agrees well with our estimate of the mitotic index by direct counting of mitotic figures in DAPI stained nuclei (Fig. 2). A lack of the expression of H3 S10 may seem inconsistent with these data. However, it has been demonstrated that the two molecular markers of mitotic cells identify different mitotic subpopulations (Juan et al. 1997). Whereas cyclin B1 is expressed throughout mitosis, docetaxel targets cells in metaphase, which is past the initiation of H3 phosphorylation in prophase (Juan et al. 1997; Prigent and Dimitrov, 2003).

In conclusion, the results of the present study support the existence of HRS in gastric and non-small cell lung cancer cell lines. Clonogenic death of gastric cancer cells by low radiation doses was significantly enhanced by a prolonged pre-treatment with low concentrations of docetaxel. In our cellular model, HRS did not correlate with the ATM/Chk1-regulated early G_2_ checkpoint arrest. Our results also suggest a new approach for the improvement the effectiveness of docetaxel-based radiotherapy of gastric cancer using low doses per fraction.

## Figures and Tables

**Table 1:** Values of the parameters in the induced repair model and the linear quadratic model fitted to the data for each cell line (AGS, A549, PC3) tested for the presence of radiation hypersensitivity in the low-dose region.

Model	AGS	A549	PC3
IR (induced repair)			
α_s_ (Gy^−1^)	1.36 ± 0.03	1.66 ± 0.04	0.88 ± 0.43
α_r_ (Gy^−1^)	0.17 ± 0.08	0.14 ± 0.02	0.26 ± 0.02
d_c_ (Gy)	0.30 ± 0.05	0.11 ± 0.03	0.14 ± 0.07
β (Gy^−2^)	0.19 ± 0.04	0.04 ± 0.00	0.06 ± 0.01
α_s_/α_r_	8.1 ± 1.1	11.5 ± 1.3	3.4 ± 1.7
LQ (linear quadratic)			
α (Gy^−1^)	0.38 ± 0.03	0.19 ± 0.02	0.33 ± 0.04
β (Gy^−2^)	0.11 ± 0.01	0.03 ± 0.01	0.04 ± 0.01
SF_2_	0.30 ± 0.01	0.60 ± 0.02	0.44 ± 0.03
